# Estimation of capture probabilities using generalized estimating equations and mixed effects approaches

**DOI:** 10.1002/ece3.1000

**Published:** 2014-03-10

**Authors:** Md Abdus Salam Akanda, Russell Alpizar-Jara

**Affiliations:** 1 Department of Mathematics, Research Center in Mathematics and Applications, University of Évora7000-671, Évora, Portugal; 2 Department of Statistics, Biostatistics & Informatics, University of DhakaDhaka, 1000, Bangladesh

**Keywords:** Closed population, generalized linear models, generalized linear mixed models, heterogeneity, population size estimation

## Abstract

Modeling individual heterogeneity in capture probabilities has been one of the most challenging tasks in capture–recapture studies. Heterogeneity in capture probabilities can be modeled as a function of individual covariates, but correlation structure among capture occasions should be taking into account. A proposed generalized estimating equations (GEE) and generalized linear mixed modeling (GLMM) approaches can be used to estimate capture probabilities and population size for capture–recapture closed population models. An example is used for an illustrative application and for comparison with currently used methodology. A simulation study is also conducted to show the performance of the estimation procedures. Our simulation results show that the proposed quasi-likelihood based on GEE approach provides lower SE than partial likelihood based on either generalized linear models (GLM) or GLMM approaches for estimating population size in a closed capture–recapture experiment. Estimator performance is good if a large proportion of individuals are captured. For cases where only a small proportion of individuals are captured, the estimates become unstable, but the GEE approach outperforms the other methods.

## Introduction

Many estimation methods have been developed for the analysis of closed population capture–recapture data. For comprehensive material on the subject see, for instance, Otis et al. (1978), Seber (2002), Williams et al. (2002) and Amstrup et al. (2005). The most general capture–recapture closed population model, considered by Otis et al. (1978) was denoted by M_tbh_ where (h) is used to denote inherent individual heterogeneity, (t) time effect, and (b) behavioral response to capture. In this work, we are interested in estimating the population size and SE of a submodel of the type M_h_, where individual heterogeneity can be modeled as a function of covariates. Development of capture–recapture models dealing with individual heterogeneity in capture probabilities has been one of the most challenging tasks. Failure to account for such heterogeneity has long been known to cause substantial bias in population estimates (Otis et al. 1978; Lee and Chao 1994; Hwang and Huggins 2005). Moreover, Link (2003) showed that without strong assumptions on the underlying distribution, estimates of population size under model M_h_ are fundamentally nonidentifiable.

The use of covariates (or auxiliary variables), if available, has been proposed as an alternative way to partially cope with the problem of heterogeneous capture probabilities (Pollock et al. 1984; Huggins 1989; Alho 1990). The idea is to model capture probabilities as a function of individual (i.e., age, sex, and weight) and environmental (i.e., temperature, rainfall, and location) covariates, using a generalized linear modeling (GLM) approach, such as logistic regression. The method of Huggins (1989, 1991), based on a conditional likelihood to estimate population size, has become very popular, but it assumes independence among capture occasions (Huggins and Hwang 2011).

In the analysis of capture–recapture data, Hwang and Huggins (2005) and Zhang (2012) examined the effect of heterogeneity on the estimation of population size by solving estimating equations, but these authors also assumed independence of capture occasions. Capture–recapture data are collected on the same individuals across successive capture occasions. One may view capture–recapture data as binary longitudinal or repeated measurements data (Huggins and Yip 2001). These repeated observations are often correlated over time. This dependency or correlation structure may be induced by incorporating individual heterogeneity. Failure to account for this dependency may provide biased estimates. Hwang and Huggins (2007) also state that the assumption of independence among capture occasions is often violated in practice, but the authors still rely on the assumption. Some dependencies among capture occasions can be dealt with through the modeling of behaviorally effects, such as trap happy and trap shy effects, which are treated as special cases in the capture–recapture literature (Yang and Chao 2005; Pradel and Sanz-Aguilar 2012). One alternative approach is to use a generalized estimating equations (GEE) to account for a working correlation structure among capture occasions (Liang and Zeger 1986) and use observed individual characteristics to model heterogeneity in capture probabilities. A mixed effects modeling approach may also be used to model heterogeneity of individual observed and unobserved characteristics in capture–recapture experiments motivating the use of generalized linear mixed models (GLMM) (Pinheiro and Bates 2000). Some authors have previously introduced the use of GLMM (logit models with normal random effects) (e.g., Coull and Agresti 1999; Stoklosa et al. 2011). An advantage of using GLMM for the estimation of capture probabilities is to accommodate not only the heterogeneity attributed to individual characteristics, but also the heterogeneity that cannot be explained by the observed individual characteristics.

Bayesian methods have also become popular in capture–recapture studies. An extensive Bayesian literature on capture–recapture closed population models includes Castledine (1981), Smith (1991), George and Robert (1992), Madigan and York (1997), Basu and Ebrahimi (2001), Ghosh and Norris (2005), King and Brooks (2008), and Gosky and Ghosh (2009, 2011). Bayesian statistical modeling requires the development of the likelihood function of the observed data, given a set of parameters, as well as the joint prior distribution of all model parameters. Bayesian methods allow for estimation of the unobserved random effects as well, but the performance of their estimates often depends on the chosen prior distributions. Often, the method of selecting prior distributions is subjective (Lee et al. 2003). A possible advantage of GEE over random-effects models and Bayesian methods relates to the ability of GEE to allow specific correlation structures to be assumed between capture occasions.

Here, we propose a GEE approach for estimating capture probabilities and population size in capture–recapture closed population studies. We also compare the results of population size estimates and their SE, when using the two estimation methodologies (i.e., GEE and GLMM). For illustrative purposes, we analyze a real data set that has already been discussed in the literature. Conditional arguments are used to obtain a Horvitz–Thompson-like estimator for estimating population size. A simulation study is also conducted to compare the performance of the estimation procedures. In the next section, we describe the notation and models that are used to estimate capture probabilities and population size.

## Notation and Models

Consider a population consisting of *N* animals in a capture–recapture experiment over *m* capture occasions, *j* = 1,2,…,*m*. Let *Y*_*ij*_ be a binary outcome, equaling 1 if the *i*th animal is being caught on the *j*th capture occasion and 0 otherwise. Let *Y*_*i*_ = (*Y*_*i*1_,*Y*_*i*2_,…,*Y*_*im*_)^′^ be a random vector with the capture history of individual *i*. Let 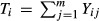 be the number of times the *i*th animal has been caught in the course of the trapping closed population study. Let *t*_*i*_ be the time the *i*th individual is first captured. Heterogeneity in captured probabilities is often explained by observed individual covariate *x*_*i*_, such as age, sex, weight. For simplicity, we consider *x*_*i*_ a single covariate, but the model can be easily generalized for *x*_*i*_ to be considered a vector of covariates. Let the probability that the *i*th animal is captured on any trapping occasion *j*, be


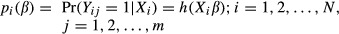
(1)

where


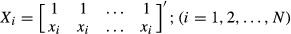


is the design matrix, *β *= (*β*_0_,*β*_1_)^′^ is the vector of parameters associated with the covariates, and *h*(*u*) = (1+exp(−*u*))^−1^ is the logistic function. This is an M_h_ model where variation in capture probabilities among individuals is explained by the covariate *x*_*i*_. The probability of not capturing the *i*th individual on the *j*th occasion is (1−*p*_*i*_(*β*)), and the variance of *Y*_*ij*_ is *p*_*i*_(*β*)(1−*p*_*i*_(*β*)) (Liang and Zeger 1986). Then, *T*_*i*_∼*Bin*(*m*,*p*_*i*_(*β*)) and *π*_*i*_(*β*) = 1−(1−*p*_*i*_(*β*))^*m*^ is the probability of individual *i* being captured at least once, given the covariate *x*_*i*_. Let the set of distinct individuals captured at least in one occasion be indexed by *i* = 1,2,…,*n* and uncaptured individuals would be indexed by *i* = *n* + 1,…,*N* without loss of generality. To estimate the population size, once an estimate of *β* is obtained (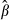), the Horvitz–Thompson estimator 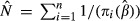 may be used as in Huggins (1989).

### Generalized estimating equations approach

Let 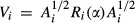 be the covariance matrix of *Y*_*i*_, where, *A*_*i*_ = diag[Var(*Y*_*i*1_),Var(*Y*_*i*2_),…,Var(*Y*_*im*_)] is a *m*×*m* diagonal matrix and *R*_*i*_(*α*) is known as the working correlation structure among *Y*_*i*1_,*Y*_*i*2_,…,*Y*_*im*_ to describe the average dependency of individuals being captured from occasion to occasion. A GEE approach permits several types of working correlation structure *R*_*i*_(*α*) (for details, see Diggle et al. 1994). For the description that follows, and for simplicity, we consider an independence working correlation structure, *R*_*i*_(*α*) = I where I is an identity matrix. The covariate *x*_*i*_ is never known for the individuals that have not been captured. Therefore, *Y*_*ij*_ is conditional on the captured individuals (*n*) (i.e., *T*_*i*_ ≥ 1) with the corresponding observed individual covariates similar to Huggins (1989) and Zhang (2012). The probability that the *i*th individual is captured on the *j*th occasion (*p*_*ij*_) given that the *i*th individual is observed at least once is, 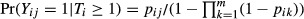. Let 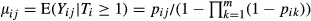, and *D*_*i*_ be the matrix of derivatives ∂*μ*_*i*_/∂*β*^′^, where *μ*_*i*_ = (*μ*_*i*1_,*μ*_*i*2_,…,*μ*_*im*_)^′^, hence *D*_*i*_ = *A*_*i*_*X*_*i*_. The variance *v*_*ij*_ of *Y*_*ij*_ given *T*_*i *_≥ 1 is 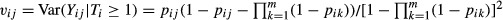. Considering, *V*_*i*_ = diag(*v*_*ij*_), an estimator of *β* can be obtained by solving the following generalized estimating equations:


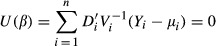
(2)

If covariate *x*_*i*_ (*i* = 1,2,…,*n*) is available for captured individuals, then the model becomes *p*_*i*_(*β*) = *h*(*X*_*i*_*β*). This model is not equivalent to any of those discussed in Otis et al. (1978), rather this model is a restricted version of their model M_h_ (Huggins 1991). If *p*_*i*_(*β*) = *h*(*X*_*i*_*β*), then following Zhang (2012), estimating equations (2) can be simplified to


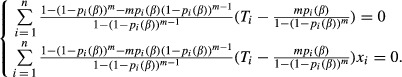
(3)

For a given 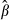, then 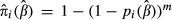 and an estimate of the variance of 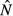 is given by 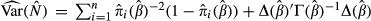 where 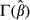 represents an estimate of the conditional information matrix for *β* and 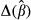 is the vector 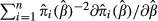. If the individual capture probability does not depend on time, previous capture history, or any covariate, then the model (1) simplifies to *p*_*i*_(*β*) = *h*(*β*_0_) = *p*_0_, which is a reparameterization of model M_0_ of Otis et al. (1978) (see Huggins 1991; Hwang and Huggins 2005). This model assumes all the individuals have equal capture probabilities. Then, the estimating equations for *β*_0_ is simplified to


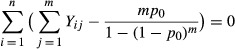
(4)

Let 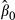 be the resulting estimator of *β*_0_ then 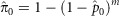 where 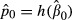.

### Methods based on a partial likelihood

The full likelihood of all model parameters is proportional to


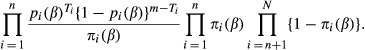
(5)

As the number of total individuals, *N*, is unknown and the covariates are not known for individuals that are never captured, this likelihood cannot be directly evaluated. The conditional likelihood (Huggins 1989) is the first product component, and it can be formulated as a GLM (Huggins and Hwang 2011) for the positive Binomial distribution (Patil 1962). It may be rewritten as


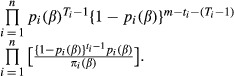
(6)

When the full likelihood is partitioned into a product of conditional densities, then a partial likelihood (Cox 1975) may arise considering some of the product terms, but it involves only the parameters of interest, isolating the nuisance parameters. Therefore, the partial likelihood, PL(*β*), is the first product of the equation (6), which is the likelihood of the number of recaptures after the first capture (Stoklosa et al. 2011). For a given *t*_*i*_, (*T*_*i*_ − 1)|*t*_*i*_∼*Bin*(*m*−*t*_*i*_,*p*_*i*_(*β*)), which is used to estimate the parameters *β*.

To utilize a simple GLMM with a random effect, we suppose that *p*_*i*_(*β*) = *h*(*X*_*i*_*β* + *σ*_*b*_*z*_*i*_) where *z*_*i*_ is a realization of the standard normal random variable 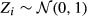, with *σ*_*b*_>0. The use of random effects reflects the belief that there is heterogeneity that cannot be explained by covariates. The partial likelihood can be considered as the joint distribution of the response and the random effects. To estimate *β* and *σ*_*b*_, the marginal likelihood of the response is obtained by integrating out the random effects. The integration can be approximated by penalized quasi-likelihood (Breslow and Clayton 1993), which enables parameter estimation via an iterative procedure.

The variance of 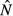 for a smoothing parameter *λ* may be estimated according to Stoklosa et al. (2011) using the following formula, 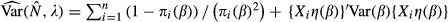, where *η*(*β*) is a vector with *η*_*i*_(*β*) = *π*_*i*_(*β*)^−2^*mp*_*i*_(*β*){1−*π*_*i*_(*β*)}, and all quantities are evaluated at 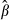. The smoothing parameter *λ*, which is part of the quasi-likelihood procedure, controls the degree of roughness of the estimated functions. To obtain an optimal value for *λ*, we used generalized cross-validation (GCV) technique (Wood 2006).

## Application

We applied the techniques discussed in the previous Section to a data set of least chipmunks (*Eutamias minimus*) made available by V. Reid (1975). The data set has been previously analyzed and discussed by Otis et al. (1978) and Wang et al. (2007). V. Reid laid out a 9 × 11 livetrapping grid with traps spaced 50 feet (15.2 m) apart. The study was conducted in an area dominated by sagebrush and snowberry in Colorado, USA. The numbers of animals caught for six occasions (*n*_1_ to *n*_6_) were 7, 15, 16, 24, 19, 7, and ∑*n*_*k*_ = 88. Of these 88 captures, *n* = 45 distinct animals were captured, and the covariate sex (male or female) was collected for each captured individual; there were 22 males and 23 females. The recorded capture frequencies (*f*_1_ to *f*_6_) were 21, 12, 7, 3, 2, 0. The average capture frequencies for male and female were 1.86 and 2.04, respectively. Our estimation results are summarized in Table 1. The inclusion of the covariate sex does not improve our estimates of population size which are very similar, except when the random effect is considered in the GLMM, which is based on partial likelihood estimation. This may indicated that there is unmodeled individual heterogeneity in capture probabilities that is not being accounted for with the other models (GLM and GEE). The population estimate, in this case, is approximately 74 individuals with a SE of 12. Both values are quite high when compared to the values obtained with the other estimation strategies. Although, GLMM accounts for heterogeneity due to unobserved individual characteristics, it may also be overestimating population size at the expenses of greater loss in precision, possibly due to the increase in the number of model parameters that are estimated. In contrast, quasi-likelihood GEE methodology provided lower SE, when compared to results from the Bayesian approach of Wang et al. (2007) for the same data set. The latter authors estimated population size of 50 with a SE of 3.14. The GEE estimation results also agree with Otis et al. (1978), but our model jointly takes into account heterogeneity in capture probabilities and correlation among capture occasions.

**Table 1:** Comparison of parameter estimates (SE in parenthesis) for least chipmunk data after fitting models with and without a covariate (sex).

Model no.	logit{*p*_*i*_(*β*)}	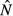
*Intercept-only models*		
1. PL GLM	−0.82 (0.18)	50.72 (3.33)
2. QL GEE	−0.73 (0.13)	49.66 (2.27)
3. PL GLMM	−0.85 (0.26) + 0.00 *z*_*i*_ (0.73)	50.73 (3.35)
*Linear covariate models*		
4. PL GLM	−0.81 (0.25) − 0.03 sex (0.37)	50.73 (3.35)
5. QL GEE	−0.84 (0.18) − 0.21 sex (0.26)	52.40 (2.94)
6. PL GLMM	−0.83 (0.34) − 0.14 sex (0.49) + 1.59 *z*_*i*_ (0.00)	74.16 (12.06)

A realization of the standard normal random variable 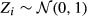 is *z*_*i*_. Numbers in this table are rounded to two decimal places; therefore, 0.00 does not mean zero.

QL, quasi-likelihood; PL, partial likelihood; GLM, generalized linear models; GEE, generalized estimating equations; GLMM, generalized linear mixed models.

## Simulation Study

A simulation study was conducted in order to evaluate the performance of the estimators. The effect of heterogeneity among observed individuals was modeled using two covariates, sex (male = 1 and female = 0), and weight. Two levels of population sizes *N* = 100 and 500 and two levels of capture occasions *m* = 6 and 10 were considered. For each individual, we assigned sex with probability 0.5 from a Bernoulli distribution and weight from a normal distribution with mean 15 and variance 4. These values are based on the previous data analysis. Individual capture probabilities were modeled with a logistic regression, so that


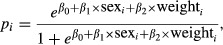
(7)

where *β*_0_ is the constant term, *β*_1_ and *β*_2_ represent the sex and weight effects, respectively. A positive *β*_1_ implies that the sex taking value 1 is more catchable, and a positive *β*_2_ means that the catchability increases with weight. We considered three different simulation scenarios for capture probabilities: (a) high capture probabilities (*β*_0_ = −3.5); (b) medium capture probabilities (*β*_0_ = −4.0); (c) low capture probabilities (*β*_0_ = −4.5); and their averaged are presented in Table 2. In addition, a Gaussian random effect with mean 0 and *σ*_*b*_ = 0.1 was included as an unobserved covariate to ensure the existence of heterogeneity due to unobserved individual characteristics. For each simulation scenario, GLM, GEE, and GLMM approaches were used for data analyses and to assess estimators performances. The simulation study was carried out with 1000 Monte Carlo replicates.

**Table 2:** Simulated capture probability scenarios for the capture probability model, logit(*p*_*i*_) = *β*_0_+*β*_1_ × sex + *β*_2_ × weight. 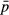 represents average capture probability when weight = 15 and *π*_*i*_ represents the average probability of an individual being captured at least once during the study.

						*π*_*i*_			
	Effects of covariates			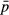		*m* = 6		*m* = 10	
Simulation scenarios	*β*_0_	*β*_1_	*β*_2_	Male	Female	Male	Female	Male	Female
(a) High	−3.5	0.1	0.2	0.40	0.38	0.95	0.94	0.98	0.98
(b) Medium	−4.0	0.1	0.2	0.29	0.27	0.87	0.85	0.94	0.92
(c) Low	−4.5	0.1	0.2	0.20	0.18	0.73	0.70	0.83	0.80

To evaluate estimators' performance, we present the SE, the relative bias (PRB), the root mean square error (RMSE), the coefficient of variation (CV), and confidence interval coverage (%) (COV) for the estimates of population size. The simulation results for six capture occasions are given in Table 3. We noticed that all estimation procedures for scenario (a) perform well. There was little bias, low SE, low coefficient of variation for 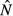. In this scenario, confidence interval coverage for all estimators is very good (93–96%), considering a nominal level of 95%. As in our example, the exception is the GLMM that tends to overestimate population size. Overestimation is particularly severe when capture probabilities are low, see for instance, results of scenarios (b) and (c). Confidence interval coverage for GLMM is also poor (77–90%) in these scenarios. For all scenarios, the GEE approach performs well when estimating population size. This approach also consistently provides lower SE and lower RMSE when compared to GLM and GLMM estimators, although the differences are minimal for GEE-GLM comparisons. Therefore, our simulation results indicate that the general performance of estimators obtained from GEE is better than GLM and GLMM. The GEE approach may overcome the effect of random effects due to its ability accounting for the correlation structure among capture occasions. The simulation results for 10 capture occasions are presented in Table 4. The performance of estimators for 10 capture occasions is better than for six capture occasions yielding lower CV, absolute value of PRB, RMSE, but higher COV. This is generally true because the average capture probability is higher for 10 capture occasions than for six capture occasions. We also conducted simulations for two other levels of *N* (50 and 200) when *m* = 6 and 10. These results are similar to the ones presented here.

**Table 3:** Simulation results (1000 repetitions) considering *m* = 6 trapping occasions.

	*N*	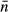	AVE( 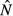 )	SE( 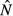 )	PRB	CV	RMSE	COV
(a) High								
PL GLM	100	92	100.63	3.77	0.63	3.75	3.82	94.5
QL GEE	100	92	100.66	2.90	0.66	2.88	2.97	95.8
PL GLMM	100	92	101.81	4.30	1.81	4.22	4.67	95.9
PL GLM	500	460	500.65	7.97	0.13	1.59	8.00	93.2
QL GEE	500	460	500.87	6.28	0.17	1.25	6.34	95.3
PL GLMM	500	460	506.56	9.07	1.31	1.79	11.20	93.1
(b) Medium								
PL GLM	100	84	101.56	7.16	1.56	7.05	7.33	94.3
QL GEE	100	84	101.51	4.82	1.51	4.75	5.05	95.2
PL GLMM	100	84	106.58	9.06	6.58	8.50	11.20	89.1
PL GLM	500	421	501.74	14.89	0.35	2.97	15.00	94.6
QL GEE	500	421	501.92	10.31	0.38	2.05	10.50	95.2
PL GLMM	500	421	526.33	18.90	5.27	3.59	32.40	83.3
(c) Low								
PL GLM	100	69	104.61	14.01	4.61	13.40	14.80	95.7
QL GEE	100	69	103.53	7.48	3.53	7.22	8.27	94.6
PL GLMM	100	69	131.07	21.14	31.07	16.10	37.60	77.2
PL GLM	500	356	504.24	26.68	0.85	5.29	27.00	95.0
QL GEE	500	356	503.86	15.45	0.77	3.07	15.90	94.5
PL GLMM	500	356	576.72	37.06	15.34	6.43	85.20	77.4

Averages of the numbers of captured individuals, (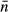); the estimates of population size, AVE(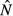); SE of the estimated population size, SE(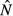); percentage relative bias, 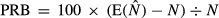, where 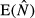 is estimated by AVE(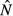; root mean square error, 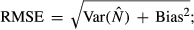; percentage coefficient of variation, 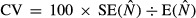 and confidence interval coverage (%), COV.

QL, quasi-likelihood; PL, partial likelihood; GLM, generalized linear models; GEE, generalized estimating equations; GLMM, generalized linear mixed models.

**Table 4:** Simulation results (1000 repetitions) considering *m* = 10 trapping occasions.

	*N*	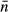	AVE( 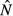 )	SE( 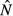 )	PRB	CV	RMSE	COV
(a) High								
PL GLM	100	98	100.11	1.43	0.11	1.43	1.43	94.3
QL GEE	100	98	100.14	1.36	0.14	1.35	1.36	96.3
PL GLMM	100	98	100.15	1.45	0.15	1.44	1.45	94.6
PL GLM	500	492	500.20	3.11	0.04	0.62	3.11	95.1
QL GEE	500	492	500.18	3.03	0.04	0.61	3.03	96.2
PL GLMM	500	492	500.28	3.19	0.06	0.64	3.20	94.9
(b) Medium								
PL GLM	100	95	100.47	3.14	0.47	3.12	3.17	95.2
QL GEE	100	95	100.42	2.98	0.42	2.97	3.01	96.5
PL GLMM	100	95	100.92	3.32	0.92	3.29	3.45	93.4
PL GLM	500	473	500.76	6.71	0.15	1.34	6.75	94.6
QL GEE	500	473	500.66	6.35	0.13	1.27	6.38	96.1
PL GLMM	500	473	502.03	7.20	0.41	1.43	7.48	94.1
(c) Low								
PL GLM	100	86	101.25	6.18	1.25	6.11	6.31	96.4
QL GEE	100	86	101.31	5.97	1.31	5.89	6.11	94.2
PL GLMM	100	86	104.71	7.35	4.71	7.02	8.73	88.6
PL GLM	500	431	500.98	13.04	0.20	2.60	13.08	95.0
QL GEE	500	431	500.65	12.57	0.13	2.51	12.58	95.4
PL GLMM	500	431	512.15	15.21	2.43	2.97	19.46	88.7

Averages of the numbers of captured individuals, (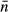); the estimates of population size, AVE(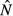); SE of the estimated population size, SE(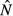); percentage relative bias, 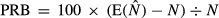, where 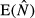 is estimated by AVE (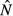; root mean square error, 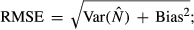 percentage coefficient of variation, 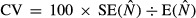 and confidence interval coverage (%), COV.

QL, quasi-likelihood; PL, partial likelihood; GLM, generalized linear models; GEE, generalized estimating equations; GLMM, generalized linear models.

## Discussion

Individual heterogeneity and time dependence are fundamentally important in real-life applications of capture–recapture studies. The main purpose of this study was to compare estimates of population size and their SE using statistical techniques such as, quasi-likelihood for GEE and partial likelihood for GLM and GLMM. We also present a GEE approach that permits capture–recapture data analysis using individual covariates that accounts for heterogeneity in capture probabilities and for correlation among capture occasions. Evaluating the pattern of time dependency is important in several regards: (i) it may help characterize the relationship between the capture probability and covariates and (ii) it is also important to estimate the population parameters accurately in the capture–recapture studies. A natural question that arise is “what happens if one ignores the time dependency and uses the traditional regression methodology assuming independence among capture occasions?” From a statistical point of view, there are at least two consequences of ignoring time dependency: incorrect assessment of the regression estimates and inefficient estimation of regression coefficients. Therefore, estimated capture probabilities may be incorrect and consequently population size may not be accurately estimated if time dependency is ignored. The quasi-likelihood GEE approach seems to perform better than GLM and GLMM approaches because the SE of the estimated population size are consistently lower. The estimators perform well when average capture probabilities are high, but it is hard to obtain reliable estimates of GLMM approach for low capture probabilities. However, other existing methods in capture–recapture studies allowing for heterogeneity have similar problems (Nichols and Pollock 1983; Nichols 1986). For cases where only a small proportion of individuals are captured, the GEE approach provides better RMSE and is robust to violation of the assumption of independence among capture occasions. This approach also provides means of exploring factors thought to be responsible for differences in capture probability among individuals. Hence, it is important to account for correlation structure among capture occasions when estimating animal population parameters in capture–recapture studies. Future work could focus on expansion of the simulations to assess the performance of estimators based on GEE, GLMM, and Bayesian methods for capture–recapture studies. Extensions of this work to model M_th_ may also be possible after imposing some parameter constraints. The GEE approach accounts for individual heterogeneity in capture probability as a function of covariates and correlation among capture occasions. It would be interesting if one can modify our proposed approach to additionally account for individual heterogeneity that cannot be explained by covariates. Researchers may also extend this approach for open population models to estimate unknown animal abundance in capture–recapture studies.

## References

[b1] Alho JM (1990). Adjusting for nonresponse bias using logistic regression. Biometrika.

[b2] Amstrup SC, McDonald TL, Manly BFJ (2005). Handbook of capture-recapture analysis.

[b3] Basu S, Ebrahimi N (2001). Bayesian capture-recapture methods for error detection and estimation of population size: heterogeneity and dependence. Biometrika.

[b4] Breslow NE, Clayton DG (1993). Approximate inference in generalized linear mixed models. J. Am. Stat. Assoc.

[b5] Castledine B (1981). A Bayesian analysis of multiple-recapture sampling for a closed population. Biometrika.

[b6] Coull BA, Agresti A (1999). The use of mixed logit models to reflect heterogeneity in capture-recapture studies. Biometrics.

[b7] Cox DR (1975). Partial likelihood. Biometrika.

[b8] Diggle PJ, Liang KY, Zeger ST (1994). Analysis of longitudinal data.

[b9] George E, Robert C (1992). Capture-recapture sampling via gibbs sampling. Biometrika.

[b10] Ghosh SK, Norris J (2005). Bayesian capture-recapture analysis of a closed population allowing for heterogeneity between animals. J. Agric. Biol. Environ. Stat.

[b11] Gosky R, Ghosh SK (2009). A comparative study of Bayesian model selection criteria for capture-recapture models for closed populations. J. Mod. Appl. Stat. Methods.

[b12] Gosky R, Ghosh SK (2011). A comparative study of Bayes estimators of closed population size from capture-recapture data. J. Stat. Theory Pract.

[b13] Huggins RM (1989). On the statistical analysis of capture experiments. Biometrika.

[b14] Huggins RM (1991). Some practical aspects of a conditional likelihood approach to capture experiments. Biometrics.

[b15] Huggins RM, Hwang WH (2011). A review of the use of conditional likelihood in capture-recapture experiments. Int. Stat. Rev.

[b16] Huggins RM, Yip PS (2001). A note on nonparametric inference for capture-recapture experiments with heterogeneous capture probabilities. Stat. Sin.

[b17] Hwang WH, Huggins RM (2005). An examination of the effect of heterogeneity on the estimation of population size using capture-recapture data. Biometrika.

[b18] Hwang WH, Huggins RM (2007). Application of semiparametric regression models in the analysis of capture-recapture experiments. Aust. NZ J. Stat.

[b19] King R, Brooks SP (2008). Bayesian estimation of a closed population size in the presence of heterogeneity and model uncertainty. Biometrics.

[b20] Lee S, Chao A (1994). Estimating population size via sample coverage for closed capture-recapture models. Biometrics.

[b21] Lee SM, Hwang WH, Huang LH (2003). Bayes estimation of population size from capture-recapture models with time variation and behavior response. Stat. Sin.

[b22] Liang K, Zeger SL (1986). Longitudinal data analysis using generalized linear models. Biometrika.

[b23] Link WA (2003). Nonidentifiability of population size from capture-recapture data with heterogeneous detection probabilities. Biometrics.

[b24] Madigan D, York JC (1997). Bayesian methods for estimation of the size of a closed population. Biometrika.

[b25] Nichols JD (1986). On the use of enumeration estimators for interspecific comparisons, with comments on a “trappability” estimator. J. Mammal.

[b26] Nichols JD, Pollock KH (1983). Estimation methodology in contemporary small mammal capture-recapture studies. J. Mammal.

[b27] Otis DL, Burnham KP, White GC, Anderson DR (1978). Statistical inference from capture data on closed animal populations. Wildl. Monogr.

[b28] Patil GP (1962). Maximum likelihood estimation for generalized power series distributions and its application to a truncated binomial distribution. Biometrika.

[b29] Pinheiro J, Bates DM (2000). Mixed-effects models in S and S-PLUS.

[b30] Pollock K, Hines J, Nichols J (1984). The use of auxiliary variables in capture-recapture and removal experiments. Biometrics.

[b31] Pradel R, Sanz-Aguilar A (2012). Modeling trap-awareness and related phenomena in capture-recapture studies. PLoS One.

[b32] Seber GAF (2002). The Estimation of animal abundance and related parameters.

[b33] Smith P (1991). Bayesian analyses for a multiple capture-recapture model. Biometrika.

[b34] Stoklosa J, Hwang W, Wu S, Huggins R (2011). Heterogeneous capture-recapture models with covariates: a partial likelihood approach for closed populations. Biometrics.

[b35] Wang X, He CZ, Sun D (2007). Bayesian population estimation for small sample capture-recapture data using noninformative priors. J. Stat. Plan Inference.

[b36] Williams BK, Nichols JD, Conroy MJ (2002). Analysis and management of animal populations.

[b37] Wood SN (2006). Generalized additive models: an introduction with R.

[b38] Yang HC, Chao A (2005). Modeling animals' behavioral response by Markov chain models for capture-recapture experiments. Biometrics.

[b39] Zhang S (2012). A GEE approach for estimating size of hard-to-reach population by using capture recapture data. Statistics.

